# CCL2/Monocyte Chemoattractant Protein 1 and Parathyroid Hormone Action on Bone

**DOI:** 10.3389/fendo.2017.00049

**Published:** 2017-03-29

**Authors:** Jawed Akhtar Siddiqui, Nicola C. Partridge

**Affiliations:** ^1^Department of Basic Science and Craniofacial Biology, New York University College of Dentistry, New York, NY, USA

**Keywords:** chemokines, monocyte chemoattractant protein 1, osteoblast, osteoclast, parathyroid hormone and bone remodeling

## Abstract

Chemokines are small molecules that play a crucial role as chemoattractants for several cell types, and their components are associated with host immune responses and repair mechanisms. Chemokines selectively recruit monocytes, neutrophils, and lymphocytes and induce chemotaxis through the activation of G protein-coupled receptors. Two well-described chemokine families (CXC and CC) are known to regulate the localization and trafficking of immune cells in cases of injury, infection, and tumors. Monocyte chemoattractant protein 1 (MCP-1/CCL2) is one of the important chemokines from the CC family that controls migration and infiltration of monocytes/macrophages during inflammation. CCL2 is profoundly expressed in osteoporotic bone and prostate cancer-induced bone resorption. CCL2 also regulates physiological bone remodeling in response to hormonal and mechanical stimuli. Parathyroid hormone (PTH) has multifaceted effects on bone, depending on the mode of administration. Intermittent PTH increases bone *in vivo* by increasing the number and activity of osteoblasts, whereas a continuous infusion of PTH decreases bone mass by stimulating a net increase in bone resorption. CCL2 is essential for both anabolic and catabolic effects of PTH. In this review, we will discuss the pharmacological role of PTH and involvement of CCL2 in the processes of PTH-mediated bone remodeling.

## Introduction

Bone remodeling is a complex process under the control of several local and systemic factors such as parathyroid hormone (PTH), vitamin D, estrogens, androgens, and inflammatory mediators. The recruitment and activation of leukocytes into the site of inflammation are necessary for inflammatory responses to infection and tissue damage. Despite the fact that leukocytes act against infection, these cells also have properties to act as growth regulators of osteoblast and osteoclast activity, suggesting their active involvement in bone metabolism. In particular, monocytes have been recognized as essential regulators of bone activity. Monocytes produce bone resorptive factors [interleukin-1 (IL-1) and tumor necrosis factor alpha (TNF-α)], which are crucial modulators of bone remodeling. Thus, leukocyte recruitment is likely to represent a notable event in both inflammation and bone metabolism.

Chemokines are small (8–15 kDa), inducible proinflammatory cytokines, characterized by the homing activity of leukocytes to targeted inflammation sites (1). Recent research indicates that chemokines participate and play divergent roles in various phases of pathogenesis and immune reactions. Chemokines have a role in the process of physiological and pathological osteoclast formation and activation. Chemokines provoke inflammation and osteoclastogenesis in pathological conditions such as arthritis- and tumor-mediated bone loss.

Several studies suggest that certain members of the CC chemokine family, including monocyte chemoattractant protein 1 (MCP-1/CCL2), macrophage inflammatory protein 1α (MIP-1α/CCL3), regulated on activation normal T-cell expressed and secreted (RANTES/CCL5), and monocyte chemoattractant protein 3 (MCP-3/CCL7), might exert their effects on committed osteoclasts.

Parathyroid hormone is a hormone synthesized and secreted by the parathyroid glands, which regulates bone mass in a conventional endocrine fashion. PTH differentially affects a number of cytokines and chemokines, such as RANKL, IL-6, CXCL1, and CCL2 (2). The expression and involvement of both CCL2 and its receptor CCR2 have been established in several pathological conditions, including rheumatoid arthritis (3), atherosclerosis (4), multiple sclerosis (5), cancer-induced bone loss (6), and bacterially induced bone loss (7). This review provides an overview of chemokines and their role in the process of physiological and pathological bone remodeling, focusing in particular on the role of CCL2 and its involvement in PTH’s actions on bone cells.

## CC Chemokines and Their Receptors

The primary functions of chemokines are to recruit monocytes, neutrophils, and lymphocytes, inducing chemotaxis by activating G-protein-coupled receptors. The chemokine family is mainly composed of four groups (CC, CXC, C, and CX3C) based on the relative position of cysteine residues. The C chemokine family has one cysteine, whereas the CC chemokine family has two adjacent cysteines near the amino terminus of the protein. The CXC and CX3C chemokine families have either one or three amino acids separating the two cysteines.

In particular, CC and CXC chemokines have a defined role in bone remodeling (8–13). The monocyte chemoattractant subfamily is a member of the CC chemokine family, which includes CCL2 (MCP-1), CCL8 (MCP-2), CCL7 (MCP-3), CCL13 (MCP-4), CCL12 (MCP-5), CCL5 (RANTES), CCL3 (MIP-1α), CCL20 (MIP-3α), and CCL4 (MIP-1β) (14–17). Among these chemokines, CCL2 is one of the most highly studied. Various cell types produce CCL2, including vascular endothelial, fibroblasts, epithelial, smooth muscle cells, astrocytic, monocytic, and microglial cells (18–21). However, principal sources of CCL2 are mononuclear leukocytes (22–24).

CCL2, originally known as JE, is a low-molecular-weight polypeptide whose primary function is to promote monocyte and macrophage migration to sites of inflammation (25). For example, CCL2 is associated with monocyte infiltration in inflammatory diseases such as rheumatoid arthritis and different tumors associated with inflammatory responses (3, 6, 7). The high-affinity CCL2 receptor, CCR2, is a member of the group of G protein-coupled receptors that contain seven transmembrane spanning domains (26). The genomic sequence of CCR2 is highly homologous and conserved through different species (27). Two alternatively spliced forms of the receptor have been identified, CCR2A and CCR2B, varying only in the *C*-terminal domain of the protein (28, 29). Although CCR2B is the predominant form, both forms of the receptor bind with high affinity to CCL2, but induce different biological responses (29). The CCR2 expression has been reported in numerous tissues, including bone, blood, brain, heart, kidney, liver, lung, ovary, pancreas, spinal cord, spleen, and thymus. It has been found that most chemokine receptors have the ability to bind several chemokines. Several reports revealed that the chemokine receptor, CCR2, could bind to five different CCL members such as CCL2, CCL7, CCL8, CCL12, and CCL13 (30–32). However, CCL2 is the most potent inducer of the signal transduction pathways leading to monocyte transmigration (33). In addition, CCL2 also binds to the CCR4 receptor, which also has CCL5 and CCL20 as ligands (34).

## CCL2/MCP-1 Chemokine and Bone Remodeling

Bone remodeling is imperative for physiological bone homeostasis. It comprises two phases: bone resorption by osteoclasts and bone formation by matrix-producing osteoblasts. The osteoblasts originate from mesenchymal stem cells in the bone marrow stroma. Osteoclasts are large, multinucleated cells formed from the fusion of mononuclear progenitors of the monocyte/macrophage in the process of osteoclastogenesis. The precise balance between bone resorption and formation is critical for the maintenance of bone mass and systemic mineral homeostasis. Any disturbance of this balance causes various bone diseases, including osteoporosis, which is characteristically defined as low bone mass and microarchitectural deterioration and extremely susceptible to fracture risk. The physiological bone remodeling process is controlled by various local and systemic factors and their expression and release in a well-organized manner. These include calcitonin, PTH, vitamin D_3_ [1,25(OH)_2_ vitamin D_3_], and estrogen. In addition to systemic hormonal regulation, other growth factors such as IGFs, TGF-β, FGFs, EGF, BMPs, Wnt family proteins, and chemokines also play a significant role in the regulation of physiological bone remodeling (35).

It has been reported that CXC and CC chemokines promote the migration of osteoclast precursor cells and facilitate the process of osteoclastogenesis and bone resorption. Study of a group of 650 patients by Eraltan et al. suggested that CCL2 and CCR2 gene variants were risk factors for osteoporosis and osteopenia (36). Graves et al. first reported that temporal and spatial expression of CCL2 by osteoblastic cells is associated with the recruitment of monocytes during inflammation and developmentally regulated bone remodeling. They also found that exogenous CCL2 enhances the recruitment of monocytes in inflamed bone (9, 37).

An early report by Volejnikova et al. showed that CCL2 is primarily expressed by bone-forming osteoblasts. They reported that the expression of CCL2 is developmentally regulated, and the recruitment of mononuclear cells in the occlusal area and basal area of the tooth is associated with bone resorption and bone formation, respectively, suggesting a differential role of monocytes in bone formation and bone resorption (38). Mechanical stresses including pressure induce chemokine (CXCL2 and CCL2) expression in osteoblasts resulting in inflammatory reactions and bone remodeling (39).

Several chemokines have been involved in different stages of osteoclastogenesis. The roles of CCL2 and its receptor CCR2 have been characterized in bone cells. The work of Rahimi et al. showed that mice with an inflammatory lesion in the mandible had elevated staining for CCL2, mainly by osteoblasts (12). RANKL stimulates the formation of osteoclasts in human peripheral blood monocyte cultures, in part, due to an increase in CCL2 production, which was shown by using blocking antibodies to CCL2 (40). CCL2-deficient mice have reduced osteoclast-specific genes (DC-STAMP, NFATc1, and cathepsin K), suggesting impaired osteoclast differentiation (41). Further, CCL2 deficiency resulted in increased bone mass and decreased bone resorption markers (CTX-1 and TRACP 5b), however, no changes in bone formation markers, suggesting that impaired osteoclastogenesis is responsible for the bone phenotype observed in CCL2 null mice (42). Studies have shown that both CCL2 and CCR2 knockout mice exhibit inadequate monocyte recruitment in response to various inflammatory conditions (1, 43–46).

It has been reported that CCR2 null mice have high bone mass and decreased osteoclast number, size, and activity (8). In osteoclast progenitor cells, CCR2 activates downstream signaling through NF-kB and ERK1/2. This publication also reported that CCR2 knockout mice develop resistance to ovariectomy-induced bone loss, suggesting the involvement of the chemokine receptor CCR2 in estrogen’s effects on bone. However, recent work by Mader et al. showed that although CCR2 null mice have larger and stronger tibiae compared to wild-type mice, they concluded that this was due to greater body mass rather than reduced bone resorption (47). In addition, they did not observe protection against ovariectomy-induced bone loss. It is worth noting that Binder et al. (8) conducted their studies on younger female mice (10–13 weeks old), while Mader et al. used 20- to 28-week-old female mice, which may account for the different conclusions of the two studies. A third report showed that CCR2 knockout mice had increased cortical BMD but less trabecular bone in both spine and distal femur (48). Recently, it has been shown that topical treatment with the CCR2 antagonist (JNJ17166864) reduced alveolar bone loss from bacterial infection in mice supporting a role for CCR2 in bone loss (49).

## Role of CCL2 in PTH Action on Bone

Parathyroid hormone is synthesized and secreted by parathyroid glands and exerts its functional role in bone mass regulation by an endocrine mode (50, 51). The PTH/parathyroid hormone-related peptide (PTHrP) receptor, also known as PTH1R, is the common receptor for both PTH and PTHrP. PTH1R is mostly expressed in bone, cartilage, and kidney cells (52–54).

Parathyroid hormone can exert both catabolic and anabolic effects on bone. It is well established that daily injections of low doses of PTH increase bone mass in animals and humans (55, 56). Continuous administration of PTH or PTHrP induces bone resorption by activating osteoclasts indirectly through their actions on osteoblastic cells (57). Several effects of PTH on osteoclast formation are mediated by stimulation of RANKL and inhibition of OPG mRNA expression (58). PTH’s anabolic effect can now be explained by evidence that PTH increases the proliferation and differentiation of osteoblasts *in vitro* and *in vivo* (59–61), decreases osteoblast apoptosis (62, 63), and activation of bone lining cells (64, 65). PTH-mediated cAMP/protein kinase A signaling is required for Runx2 transactivation, which in turn upregulates the expression of osteoblast genes. In addition, intermittent PTH also activates ERK1/2-mitogen-activated protein kinase and phosphatidylinositol phosphate signaling pathways, resulting in increased osteoblast proliferation (66, 67).

The PTH1R exists predominantly on osteoblasts, osteocytes, and preosteoblast-like cells. It has been well established that osteoblast-secreted factors play an essential role in PTH-mediated osteoclastic bone resorption. M-CSF and RANKL are two well-known factors necessary for proliferation of osteoclast progenitors and their differentiation into mature osteoclasts (68, 69). Kim et al. have shown that CCL2 promotes human osteoclast fusion (40, 70). These authors have also reported that co-treatment of peripheral blood mononuclear cells with CCL2 and M-CSF stimulates their differentiation toward osteoclast-like cells even in the absence of RANKL (70). By contrast, Li et al. (11) have shown that CCL2 alone does not have the ability to differentiate mouse BMMs to mature osteoclasts. But CCL2 accompanied by RANKL highly augments RANKL’s effect on osteoclastogenesis (11). Kim et al. used human peripheral blood mononuclear cells, whereas Li et al. used mouse bone marrow cells. Perhaps the use of these cells of different origins and different duration of culture are factors in the differences in findings. There is also the possibility that the human peripheral blood mononuclear cells have been already exposed to RANKL. Nevertheless, both studies suggest that CCL2 has a role in the activation of bone resorption.

Intermittent PTH increases bone formation and promotes bone remodeling (71). We have shown that CCL2 is the most highly upregulated gene in rat femurs 1 h after the 14th daily hPTH(1–34) injection (2, 11), with nearly 200-fold stimulation of its mRNA expression. Osteoclasts and monocytes are likely to be the central targets for CCL2 in bone. PTH-induced osteoblastic expression of CCL2 facilitates osteoclast recruitment, differentiation, and fusion of osteoclast precursors and finally provides a rationale for increased osteoclast activity in the anabolic effect of PTH (11). We reported a significant increase in serum CCL2 levels 2 h after PTH injection compared with basal levels in rats treated daily with hPTH(1–34) (13). We also found a profound increase in CCL2 expression in osteoblasts *in vivo* by immunohistochemistry and *in vitro* in UMR 106-01 and rat primary calvarial osteoblastic cells after PTH treatment (11). As well, CCL2 null mice were completely unable to increase trabecular BMD and bone volume compared to wild-type mice after daily injections of PTH. In addition, these mice did not show the increase in macrophage numbers, osteoclast surface, and osteoclast number observed in wild-type mice after PTH injections. We concluded that the reduction in PTH-mediated bone formation in CCL2 null mice was due to the lack of osteoclast and macrophage activity and that osteoblast CCL2 expression is a key mediator for the anabolic effects of PTH on bone (13).

**Figure 1:**
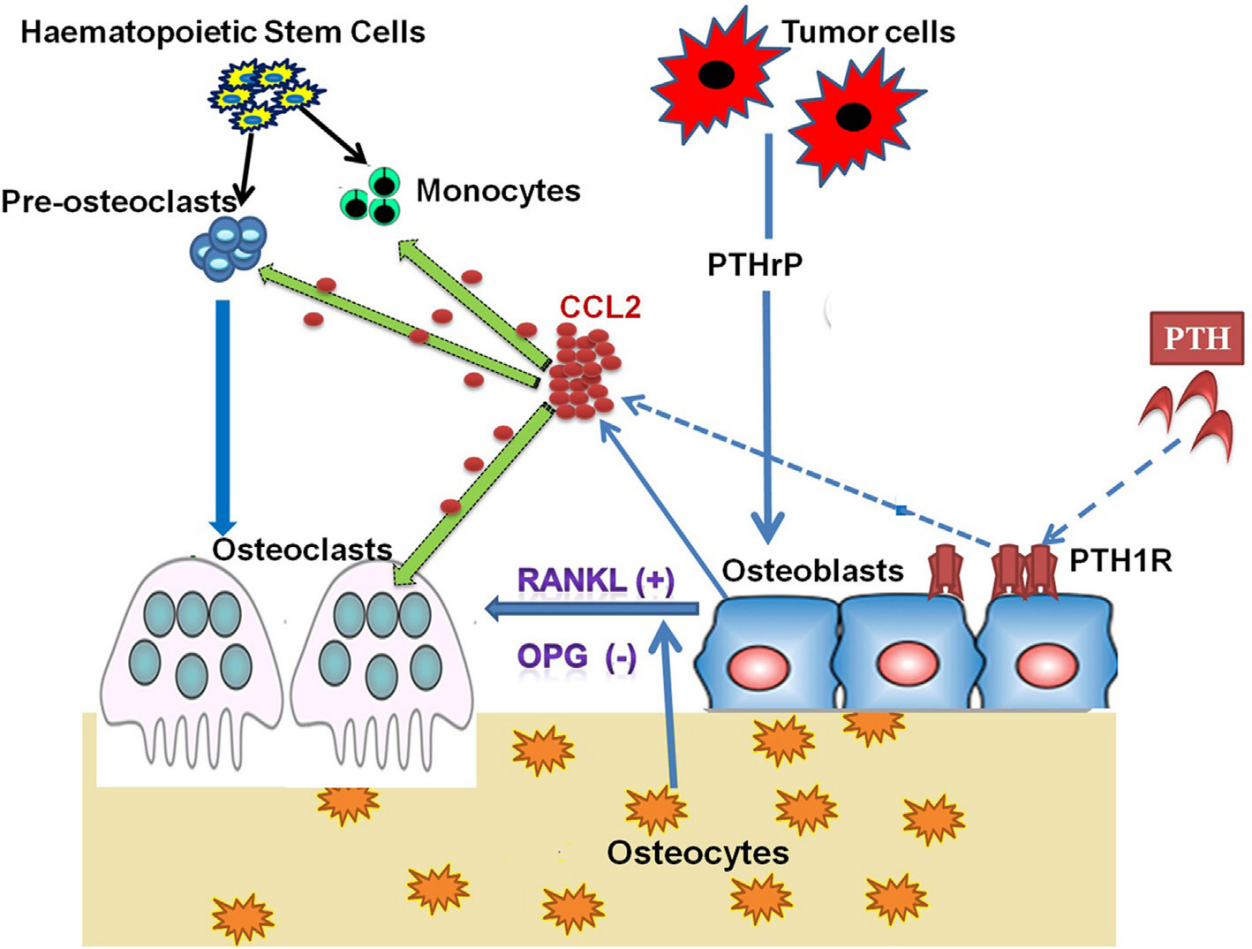
**Role of CCL2 in PTH/PTHrP Action on Bone Cells**. Osteoclasts are large, multinucleated cells formed from the fusion of mononuclear progenitors of the monocyte/macrophage lineage. However, osteoblasts originate from mesenchymal stem cells. In the process of bone remodeling, both osteoblasts and osteoclasts are highly dependent on one another to sustain normal bone mass. Osteoblasts secrete CCL2 on binding parathyroid hormone (PTH) to its receptor parathyroid hormone/parathyroid hormone-related peptide receptor (PTH1R), which is present on the osteoblast. PTH-induced CCL2 facilitates the recruitment of monocytes and preosteoclasts to remodeling sites. At the same time, CCL2 also participates in the fusion of preosteoclasts to mature osteoclasts. The transient increase in CCL2 expression and resultant osteoclast activity is required for the anabolic effect of PTH on bone. Tumor cells produce parathyroid hormone-related peptide (PTHrP), which stimulates CCL2 expression from bone-forming osteoblasts. Osteoblastic CCL2 increases osteoclastogenesis and bone resorption to facilitate tumor growth in bone.

It has been reported that inhibitors of bone resorption such as bisphosphonates can blunt the PTH-mediated osteoanabolic effect, signifying the role and requirement of active bone resorption for the anabolic actions of PTH (72, 73). Intermittent PTH causes transient upregulation of CCL2 and RANKL, which initiates bone resorption that ultimately increases net bone formation. Collectively, PTH treatment activates the PKA pathway in osteoblasts, which increases the expression and secretion of CCL2 and expression of RANKL by osteoblasts. CCL2 facilitates the recruitment of osteoclasts and its precursor monocytes for bone remodeling (Figure 1). Simultaneously, CCL2 also assists the fusion of the preosteoclast to form mature multinucleate osteoclasts (11).

By contrast, with continuous infusion of PTH in a catabolic protocol in rats, we found that both RANKL and CCL2 expression were moderately increased, and this was sustained throughout the period of PTH treatment. The constant elevated levels of CCL2 may enhance RANKL-mediated osteoclastic bone resorption (11). This contrasts with the very transient but high upregulation of CCL2 by daily injections of PTH. It is very noticeable that there is a progressive and adaptive response to PTH in the anabolic protocol, and this may be a key difference distinguishing the role of CCL2 in catabolic versus anabolic effects of PTH. We think that the difference in the kinetics of gene expression, RANKL and CCL2, are responsible for the net increase in bone mass in the anabolic protocol compared with the net increase in bone resorption in the catabolic protocol.

Some recent clinical studies have shown a positive relationship between PTH and MCP-1 levels. Sukumar et al. found a positive association between serum levels of CCL2 and PTH in women with primary hyperparathyroidism (PHPT) (74). Elevated CCL2 levels increase the risks of hypertension, hyperlipidemia, type 2 diabetes mellitus, and coronary artery disease. A small clinical study by Patel et al. also showed that an immediate decline in high serum CCL2 concentrations after parathyroidectomy of PHPT patients further prove the positive association between MCP-1 and PTH levels in patients with PHPT (75).

Parathyroid hormone-related peptide is a genetically related peptide that shares homology with PTH within its amino-terminal domain, which is considered to contain the essential bioactivity of PTH and thus could mimic several functions of PTH (50). PTHrP is synthesized in bone and cartilage and exerts its functions in autocrine and paracrine modes. It has been shown that PTHrP augments bone metastasis in animal models of both prostate cancer and breast cancer (76, 77). Li et al. showed that synthetic PTHrP increased secretion of CCL2 from osteoblastic cells. Further, prostate cancer cell lines (PC-3 and VCaP) elevated bone marrow CCL2 levels in a mouse xenograft model. In line with these findings, they found an increase in CCL2 levels from osteoblastic cells cultured with conditioned medium from the same high PTHrP-secreting prostate cancer cell lines. Treatment with a neutralizing antibody to CCL2 decreased tumor burden and bone resorption (6). In another study, the authors found that autocrine and paracrine functions of CCL2 are required for prostate cancer growth and invasion, and treatment with a CCR2 antagonist reduced the CCL2-mediated prostate cancer growth and invasion (78). Taken together, PTHrP enhances CCL2 expression in osteoblasts, signifying the role of prostate cancer cell-derived PTHrP in bone metastasis, likely *via* enhanced CCL2 expression (6, 79, 80). These studies suggest that the CCL2/CCR2 axis might be a potential therapeutic target for prostate cancer-bone metastasis treatment.

Regulation of CCL2 has investigated its promoter region, which consists of two C/EBP binding sites, two NF-kB binding sites, and a GC box. The C/EBP binding sites, NF-kB binding sites, and GC box are important for the response to insulin activation, IL-1 and TNF-α activation, and SP1 binding, respectively. Very little is known of how PTH regulates the CCL2 promoter in osteoblastic cells, and it remains a mystery since this is a primary response gene regulated by the PKA pathway, yet there is no obvious CRE in the upstream region. It has been stated that PTHrP provokes CCL2 promoter activity in hFOB cells through NF-kB and C/EBP activation (80), but none has established how the PKA pathway can regulate these factors to stimulate CCL2 transcription.

## Conclusion

Bone remodeling is a complex process under the control of several factors, including hormones, growth factors, and other inflammatory mediators. Among them, chemokines and their particular receptors play a vital role as chemoattractant and growth factors for bone cell recruitment and regulation of osteoclastogenesis, respectively.

Chemokines recruit and activate leukocytes at the site of inflammation in response to infection or tissue damage. These leukocytes are primary cells responsible for inflammatory responses and bone metabolism, since they are capable of acting as growth regulators of both osteoblast and osteoclast activity. Growing evidence suggests that CCL2 and its receptor CCR2 are involved in the physiological bone remodeling process. *In vitro* and *in vivo* models, together with both ligand and receptor transgenic animals, have made a significant contribution to understanding the molecular mechanisms behind the role of CCL2 in PTH-mediated bone effects.

A more comprehensive understanding of the chemokines that regulate bone remodeling could reveal new possibilities for the development of novel and more successful drug therapies for the treatment of severe bone loss diseases, including osteoporosis, rheumatoid arthritis, or cancer-mediated bone metastasis. In conclusion, modulation of the CCL2/CCR2 axis may provide the potential mechanism to therapeutically limit the bone resorption and blunt bone loss.

## Author Contributions

JS drafted and wrote the manuscript, and NP reviewed and finalized the manuscript.

## Conflict of Interest Statement

The authors state that they have no conflicts of interest pertaining to the work described.
